# Commentary: Expression of salivary hepcidin and its inducer, interleukin 6 as well as type I interferons are significantly elevated in infants with poor oral rotavirus vaccine take in South Africa

**DOI:** 10.3389/fimmu.2025.1651751

**Published:** 2025-08-12

**Authors:** Artur Słomka

**Affiliations:** ^1^ Department of Pathophysiology, Nicolaus Copernicus University in Toruń, Ludwik Rydygier Collegium Medicum in Bydgoszcz, Bydgoszcz, Poland; ^2^ Department of Digital Medicine, Implementation & Innovation, National Medical Institute of the Ministry of Interior and Administration, Warsaw, Poland

**Keywords:** hepcidin, iron, saliva, vaccination, rotavirus

## Introduction

With great interest, I read the article by Mabasa and colleagues, published over a month ago in *Frontiers in Immunology* (Mabasa, V., Seheri, M. L., & Magwira, C. A. (2025). Expression of salivary hepcidin and its inducer, interleukin 6 as well as type I interferons are significantly elevated in infants with poor oral rotavirus vaccine take in South Africa. Frontiers in immunology, 16, 1517893.) (1). The authors demonstrated a potential relationship between disturbances in iron metabolism and the efficacy of rotavirus vaccination (1). Given the significance of rotavirus infections, particularly in developing countries, such observations, as presented by the researchers from South Africa, are especially valuable (2). It is worth emphasizing that the association between iron and the inflammatory process is well established. However, in the context of viral diseases, there is no clear consensus regarding their relationship with iron homeostasis, and reported effects are often heterogeneous or even contradictory (3). Considering the clinical significance of rotavirus infection and the potential interplay between iron homeostasis and mucosal immunity, the investigation of this topic appears well-justified and warrants further scientific exploration.

## Critical appraisal of methodological considerations and inherent limitations in the discussed study

Although Mabasa et al. (1) deliver valuable preliminary evidence elucidating the intricate relationship between iron homeostasis disruptions and immunogenic responses elicited by rotavirus vaccination, several methodological constraints inherent to their study must be meticulously scrutinized. As appropriately noted by Mabasa and colleagues, these limitations impose critical caveats on the clinical interpretability and generalizability of the observed associations.

### Considerations regarding biomarker assay validation and biological sample specificity

The authors based their observations on hepcidin, a protein described approximately twenty-five years ago, which acts as a cellular regulator by inhibiting the absorption and release of iron in response to its excess (4). Among other measurements, the authors assessed the concentration of hepcidin in the saliva of infants who had received the rotavirus vaccine. Although the authors do not explicitly justify their choice of saliva as the biological matrix, it may be reasonably presumed that this decision was influenced by practical considerations related to the age and clinical context of the studied population. Nonetheless, it should be noted that salivary hepcidin testing is infrequent and remains insufficiently validated, making it difficult to formulate sound hypotheses or draw definitive conclusions. Although hepcidin is detectable in saliva (**Table 1**), the relationship between this salivary fraction and systemic hepcidin levels is entirely unknown. The mechanisms regulating salivary hepcidin concentration and the extent to which it reflects the biological properties of the protein at the systemic level remain unclear.

**Table 1:** Summary of selected studies assessing salivary hepcidin concentration in various clinical contexts.

First author, year	Study population	Number of participants	Age, years (Mean ± SD)	Sex of participants (M/F)	Hepcidin assay	Salivary hepcidin concentration [ng/mL] (Mean ± SD or Median [IQR])	Key findings
Arnold, J., 2010 (5)	Healthy individuals	17	35 ± 9.9	8/9	RIA	3.39 ± 2.83	Hepcidin is detectable in saliva
Cicek, D., 2014 (6)	Behçet’s disease (BD)	25	34.48 ± 6.9	13/12	ELISA	657.58 ± 358.25	Increased hepcidin concentration in RAS patients
	Recurrent aphthous stomatitis (RAS)	30	31.56 ± 11.5	15/15		443.10 ± 249.68	
	Healthy individuals	25	31.52 ± 6.2	13/12		714.10 ± 280.58	
Guo, L. N., 2018 (7)	Chronic periodontitis (CP)	22	58.09 ± 9.97	16/6	ELISA	1.64 [0.93, 3.19]	No significant differences between study groups
	Type 2 diabetes mellitus (T2DM)	22	56.45 ± 11.80	15/7		1.54 [0.99, 3.80]	
	CP+T2DM	22	62.82 ± 10.72	17/5		1.79 [0.93, 5.14]	
	Healthy individuals	22	52.45 ± 10.01	14/8		1.12 [0.89, 1.73]	

BD, Behçet’s disease; CP, Chronic periodontitis; ELISA, Enzyme-linked immunosorbent assay; F, Female; IQR, Interquartile range; M, Male; RAS, Recurrent aphthous stomatitis; RIA, Radioimmunoassay; SD, Standard deviation; T2DM, Type 2 diabetes mellitus.

Prior investigations undertaken by other researchers have predominantly explored salivary hepcidin concentration across diverse cohorts of patients and healthy individuals, primarily with the objective of elucidating oral and dental pathological alterations (5–7). These observations are partially corroborated by limited evidence derived from animal studies (8). It warrants particular attention that investigations into salivary hepcidin remain in a nascent and fragmentary stage, frequently constrained by limited cohort sizes and a lack of methodological harmonization. As illustrated in **Table 1**, the reported concentrations of salivary hepcidin exhibit striking heterogeneity, which may stem not only from divergent clinical scenarios or demographic variables but also from discrepancies in sample acquisition protocols, pre-analytical processing, and the analytical platforms employed. A particularly illustrative example is the comparison between the studies by Cicek et al. (6) and Guo et al. (7), both of which assessed salivary hepcidin in healthy individuals using immunoenzymatic assays, yet reported values that differ by over two orders of magnitude. Such pronounced variability unequivocally highlights the current absence of stringent methodological control in this emerging area of biomarker research. Given the paucity of comprehensive validation and the uncertain interpretative value of salivary hepcidin in the context of systemic iron regulation, it may be more methodologically prudent to consider alternative salivary biomarkers such as iron or ferritin concentrations, both of which have been subjected to more extensive analytical evaluation and are supported by a more robust body of evidence concerning their clinical relevance within the salivary matrix (9–11).

In light of the scarce and heterogeneous data on salivary hepcidin, I contend that its selection as an analytical matrix, while presumably driven by the need for non-invasive sampling in pediatric settings, remains insufficiently justified. This observation leads me to the conclusion that a well-founded scientific and clinical rationale is essential before employing this biomarker in infant research.

### Constraints in sample size and inferential strength

Mabasa et al. (1) conducted their investigation in a cohort of 121 Black neonates who received the rotavirus vaccine at six weeks of age. While the authors characterize their study as cross-sectional in nature, this designation appears methodologically imprecise. In classical epidemiological terms, a cross-sectional design entails the assessment of variables at a single, defined time point. However, in this study, biological samples were collected at both six and seven weeks of age, thereby introducing a temporal component inconsistent with strict cross-sectional methodology.

At these two time points, the investigators obtained unstimulated saliva and stool samples to quantify hepcidin concentration and expression, evaluate the transcriptional activity of inflammatory molecular regulators, and determine vaccine shedding status (i.e., shedders vs. non-shedders). A point of particular concern arises from the substantial attrition in salivary sampling: although 121 neonates were initially enrolled, saliva was ultimately collected from only 48 participants - 30 shedders and 18 non-shedders. The authors provide no clarification regarding the exclusion or loss of the remaining individuals, leaving a critical gap in the methodological transparency of the study.

Such a markedly reduced sample size raises questions about the statistical validity of the analyses performed, particularly with respect to subgroup comparisons. Equally notable is the absence of a formal power analysis, which would have enabled the authors to determine the minimum sample size required to detect meaningful differences with an acceptable risk of Type II error. Insufficient statistical power inherently compromises the inferential strength of the findings and increases the likelihood of false-negative results, potentially obscuring clinically relevant associations.

## Interpretative uncertainties surrounding hepcidin-related findings

The primary findings reported by Mabasa et al. concern the absence of statistically significant differences in salivary hepcidin concentration between vaccine shedders and non-shedders at both six and seven weeks of postnatal life. Notwithstanding this overall uniformity, a particularly thought-provoking observation arises from the intra-group analysis, which demonstrated a temporal decrease in salivary hepcidin concentration in both subpopulations at the second sampling point.

This finding, however, gives rise to a series of fundamental mechanistic and interpretative uncertainties, particularly when considered against the backdrop of the study’s pronounced methodological shortcomings. Crucially, it remains unclear whether the observed reduction reflects a localized immunometabolic response or a broader systemic disturbance in iron homeostasis. Equally speculative is the question of whether this phenomenon is causally related to vaccine-induced immune activation or merely represents an incidental, physiologically irrelevant fluctuation. In the absence of mechanistic correlates and a more rigorous study design, the clinical and biological significance of this observation remains indeterminate.

The authors’ hypothesis suggesting a potential association between vaccine shedding status) and hepcidin concentration is, therefore, questionable. While the effect of vaccination itself on hepcidin levels may be conceptually justified within the context of the study, the authors did not convincingly establish a rationale for investigating the relationship between salivary hepcidin and the presence of vaccine-derived virus in the stool of infants. It is also worth emphasizing that the authors may have misinterpreted certain aspects of their findings. This pertains not only to the questionable conclusions drawn with regard to iron deficiency anemia (IDA), a matter discussed in greater depth in a subsequent subsection, but also to a more fundamental issue: both the enzyme-linked immunosorbent assay (ELISA) and polymerase chain reaction (PCR) analyses ultimately produced concordant results, indicating no discernible differences in salivary hepcidin concentration or gene expression between shedders and non-shedders. Stated differently, by the seventh day post-vaccination, both salivary hepcidin concentration and gene expression appeared indistinguishable between neonates with and without detectable rotavirus shedding in stool specimens. This finding, therefore, ought to be interpreted accordingly: the lack of divergence in these hepcidin-related parameters fundamentally undermines its utility as a biomarker, both from a mechanistic perspective and within a translational or clinical framework. Consequently, the clinical relevance and internal logic of this aspect of the study appear to be limited.

## Ancillary methodological reflections and limitations

My concern also extends to the rather liberal approach taken by the authors in diagnosing iron deficiency anemia (IDA). In this regard, they relied solely on hepcidin concentration, despite the fact that even serum hepcidin, much better characterized, remains of limited diagnostic utility due to various confounding factors, particularly inflammation (12). It is therefore surprising that the authors did not employ more widely accepted laboratory parameters routinely used to assess iron status. The diagnosis of IDA was based on criteria proposed by Prentice and colleagues (13), which had been applied by them specifically to evaluate iron supplementation in children, with hepcidin concentrations measured in blood. Accordingly, the authors’ reasoning for using salivary hepcidin to diagnose IDA appears fundamentally flawed and unsupported by any empirical evidence or established diagnostic practice.

It would also have been of interest to explore potential associations between the demographic and clinical characteristics of the infants and their mothers and various laboratory parameters. Although certain demographic data were provided, they were not included in the analytical section of the study. An analysis of this kind, which could have been performed using routinely collected clinical information, would have strengthened the overall findings, especially given the limited insight offered into the characteristics of the study population.

## Discussion

This General Commentary offers a critical appraisal of a recently published investigation into the purported role of salivary hepcidin as a biomarker of oral rotavirus vaccine immunogenicity in infants. The referenced study, while addressing a clinically relevant and timely intersection between mucosal immunology and iron metabolism, presents notable methodological and conceptual limitations that necessitate a more nuanced and rigorous scientific discourse.

From a critical standpoint, the principal conclusion of the study ought to have emphasized the observed decline in salivary hepcidin concentration following vaccination, which, notably, appears to be independent of the infants’ shedding status. Importantly, the authors did not establish any mechanistic or clinically substantiated association between their findings and IDA. Consequently, the assertion in the abstract regarding a putative “impediment” of IDA in the context of rotavirus vaccination represents a conceptual overstatement that is not supported by the underlying biological or clinical evidence. This claim warrants careful reconsideration in light of the study’s methodological omissions and evident overinterpretation of statistical outcomes.

Herein, I have sought to offer a constructive critique of a study that touches on an important intersection between iron homeostasis and mucosal immunization. While the research question itself is of high clinical relevance, I believe that several methodological oversights, questionable interpretative steps, and insufficient analytical depth limit the strength of the conclusions drawn. Future investigations would benefit from validated biomarkers, multidimensional clinical correlations, and greater precision in defining both exposures and outcomes. Such rigor is essential if we are to fully understand the complex interplay between micronutrient metabolism and vaccine performance in early life.
